# Effects of dietary glycerol monolaurate supplementation on milk production and methane emissions in Holstein dairy cows

**DOI:** 10.3168/jdsc.2024-0567

**Published:** 2024-09-06

**Authors:** R.L. Culbertson, P. Uzun, N. Seneviratne, A.B. Portela Fontoura, A.N. Davis, J.W. McFadden

**Affiliations:** 1 Department of Animal Science, Cornell University, Ithaca, NY 14853; 2 Department of Food Technology, Isparta University of Applied Sciences, Isparta, Turkey 32200; 3 Department of Biological Sciences, State University of New York Cortland, Cortland, NY 13045

## Abstract

Glycerol monolaurate (GML) has antimicrobial and anti-inflammatory properties in nonruminants. In vitro, GML reduces methane (CH_4_) production; however, the effects of dietary GML supplementation on milk production and CH_4_ emissions have not been evaluated in dairy cattle. In a completely randomized design, 42 mid-lactation Holstein cows (3.10 ± 1.08 lactations; 40.0 ± 6.65 kg milk/d) were acclimated to a tiestall barn for 3 wk and assigned to 1 of 3 treatments (n = 14/treatment): unsupplemented (CON), low-dose GML (50 g/d; LD), or high-dose GML (150 g/d; HD) for a 21-d experimental period. Cows were fed a base diet composed of corn silage, grass haylage, and concentrates and milked thrice daily. During the final week of acclimation and the experimental period, milk was sampled consecutively for 9 milkings/week. Methane, carbon dioxide (CO_2_), and hydrogen (H_2_) gas measurements were collected 3 times per day over 4 d (12 samples/cow) using the GreenFeed system (C-Lock Inc., Rapid City, SD). The statistical model included fixed effects of treatment, day, and treatment × day as well as random effect of cow. Planned contrasts to compare CON versus LD and CON versus HD were employed. Dry matter intakes, milk yields, and ECM yields were not affected by treatment. However, HD cows tended to produce more 3.5% FCM, relative to CON (46.6 vs. 44.8 kg/d). Milk fat contents (4.22% vs. 4.01%) and yields (1.76 vs. 1.62 kg/d) were greater in HD, relative to CON. The LD and HD cows had higher contents and yields of de novo fatty acids, including lauric acid, relative to CON. Milk protein contents were lower for HD cows, relative to CON (3.42% vs. 3.49%). Milk protein yields were lower in LD and HD, relative to CON (1.46, 1.46, and 1.52 kg/d, respectively). Milk lactose contents and yields were not affected by treatment. Cows provided the HD treatment tended to have improved feed efficiency (i.e., kg of milk, 3.5% FCM, and ECM per unit of DMI), relative to CON. Methane production, intensity, and yield, and CO_2_ and H_2_ production were not affected. We conclude that dietary GML supplementation altered milk production; however, GML feeding at the levels used in this study did not modify enteric CH_4_ production, intensity, or yield in dairy cattle.

## Highlights

• Dietary GML supplementation increased milk fat contents and yields.
• Dietary GML supplementation altered milk fatty acid profiles.
• Dietary GML supplementation lowered milk protein contents and yields.
• Dietary GML supplementation had no effect on yields of milk or energy-corrected milk.
• Dietary GML supplementation had no effect on methane, carbon dioxide, or hydrogen production.

## Graphical Abstract

**Figure:**
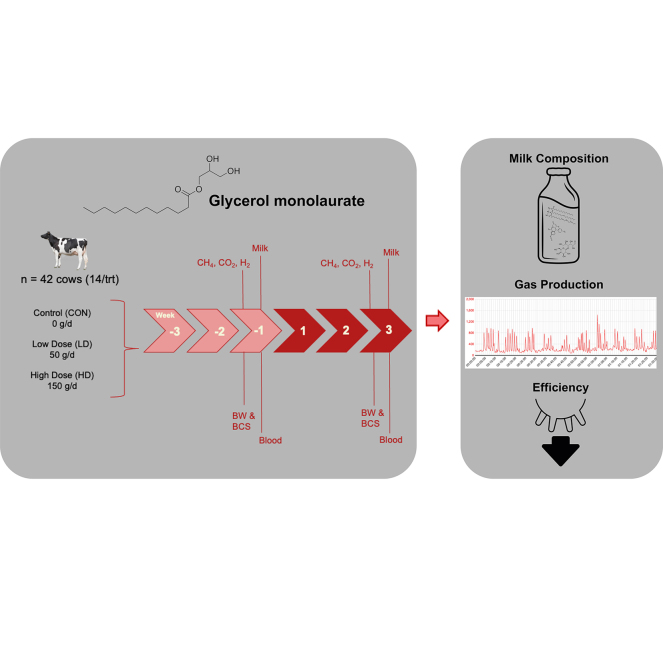
**Summary:** Glycerol monolaurate (GML) feeding provokes changes in immune function and performance in nonruminants. In bovine rumen fluid, GML alters fermentation patterns and reduces methane production. Our objective was to determine whether dietary GML supplementation modifies milk production and ruminal methane emissions. Mid-lactation Holstein cows (n = 42) were assigned to 1 of 3 treatments (trt): unsupplemented (CON), low-dose GML (50 g/day; LD), or high-dose GML (150 g/day; HD) for 21 days. Milk yields and energy-corrected milk yields were not modified. Milk fat contents and yields were greater in HD relative to CON. The LD and HD cows had higher concentrations and yields of de novo fatty acids, including lauric acid. Milk protein yields were lower in LD and HD relative to CON. Methane production, intensity, and yield, and carbon dioxide and hydrogen production were not modified. Short-term GML feeding modified milk production, but not gas emissions from lactating cows.

The monoglyceride monolaurin, also known as glycerol monolaurate (**GML**), is the monoester formed from glycerol and lauric acid. Previous work has established the benefits of dietary GML supplementation on the immune system, optimizing the intestinal microbiome and antioxidant balance in broilers (Kong et al., 2021), inhibiting African Swine Fever virus, (Jackman et al., 2020), and preventing inflammation in human epithelial cells (Schlievert et al., 2019). We lack understanding of how GML modifies milk and methane production in lactating cows.

Ruminants have been identified as contributors to anthropogenic greenhouse gas emissions, including methane (CH_4_). Methanogenesis in ruminants not only poses climate challenges, but it also represents energy from intake that is not used for maintenance, growth, or lactation (Chagunda et al., 2009). One possible enteric CH_4_ mitigation strategy is dietary medium-chain fatty acid (**FA**) supplementation. Lauric acid (C12:0) and myristic acid (C14:0) are 2 medium-chain FA that effectively inhibit CH_4_ production (Dohme et al., 2001). Lauric acid is most active against microbes when it is esterified with glycerol to form GML (Kabara, 1984). Glyceridic lipids undergo rapid hydrolysis within the rumen, leading to the release of glycerol and FA (Garton, 1969). In the case of GML, the glycerol backbone is fermented and lauric acid is available in the rumen. In vitro, GML decreased CH_4_ production 32% in a maize-based diet at a dose of 50 g/kg DM (Klevenhusen et al., 2009). The mode of action is still in question, though it is believed GML can suppress archaea, lower protozoal counts, shift fermentation toward propionate production, and hinder fiber fermentation (Klevenhusen et al., 2009); however, a potential concern is that high feeding levels of lauric acid, and potentially GML, may reduce DMI (Klop et al., 2017). We hypothesized that dietary GML supplementation would decrease enteric CH_4_ emissions and improve milk production and composition because of greater energy available to the animal or a shift in rumen fermentation toward propionate. Our objective was to evaluate the effects of dietary GML supplementation on milk yield, composition, and CH_4_ production in lactating cows.

All experimental procedures were conducted in accordance with the Cornell University Institutional Animal Care and Use Committee (protocol no. 2022–0216; Ithaca, NY). Forty-two multiparous, mid-lactation Holstein cows (3.10 ± 1.08 lactations; 40.0 ± 6.65 kg milk/d) were enrolled in a study with a completely randomized design at the Cornell Dairy Research Center (Harford, NY). Following a 3-wk acclimation to the tiestall barn, and training to the GreenFeed units (C-Lock Inc., Rapid City, SD), cows were assigned to 1 of 3 treatment groups (14 cows/treatment): the basal diet (**CON**), the basal diet plus a low dose of GML (50 g/d; **LD**), and the basal diet plus a high dose of GML (150 g/d; **HD**). Considering the molecular weight of the glycerol backbone, the LD group received ∼36.5 g/d of lauric acid and the HD group received ∼109.5 g/d of lauric acid. These doses were chosen to avoid reductions in DMI, which have been observed with cows fed lauric acid at a much higher dose (65 g/kg of DM; Klop et al., 2017). The potential cost to the producer were also considered. As such, this study serves to evaluate the effects of GML supplementation under a real-world dairy cattle management scenario, and the chosen dose aims to strike a balance between practical feasibility, cost effectiveness for farmers, and the avoidance of potential of negative impacts on DMI that may jeopardize animal performance. Treatment groups were balanced by milk yield, DIM, and parity at assignment. Diets were predominantly composed of corn silage, grass haylage, ground corn, soybean meal, soy hulls, molasses, and vitamins and minerals (Table 1). All diets were formulated using AMTS.Farm.Cattle(Pro) using a model second-lactation cow with a BW of 709 kg, producing 45.0 kg milk/d, with 4.00% milk fat and 3.00% milk protein, estimated to consume 26.8 kg DM/d. The GML was provided as a mix with concentrates, and control cows received a mix containing only concentrates (Table 1). Cows were fed mixed rations in accordance with standard farm management at ∼0700 h and milked at 0600, 1400, and 2200 h daily. Individual feed, TMR, and orts samples were collected weekly for DM analysis and composited for chemical analysis. Milk yields and feed intakes were recorded daily. Body weights and BCS were measured during the final week of acclimation (wk −1) and the experimental period (wk 3).

**Table 1:** Nutrient composition (% of DM unless otherwise noted) of experimental diets fed to multiparous lactating Holstein cows

Item	Treatment			
	Acclimation	CON^1^	LD^2^	HD^3^
Ingredient, %				
Corn silage	49.4	49.0	49.0	49.0
Grass haylage	11.4	11.3	11.3	11.3
Concentrate mix A^4^	39.2	38.8	38.8	38.8
Concentrate mix B^5^	—	0.86	—	—
Concentrate mix C^6^	—	—	0.86	—
Concentrate mix D^7^	—	—	—	0.86
Nutrient composition, %				
DM	47.7	47.8	48.0	47.7
CP	15.7	16.2	16.2	15.4
aNDFom	31.8	31.9	29.9	32.5
ADF	19.2	18.3	17.5	18.9
Starch	25.5	25.1	24.3	25.3
Sugar	3.70	3.70	3.10	2.60
Crude fat (EE)	4.04	4.14	4.41	5.01
Ash	6.83	7.10	7.19	6.53
Calcium	0.81	0.86	0.87	0.70
Phosphorus	0.38	0.38	0.38	0.37
Magnesium	0.28	0.29	0.29	0.28
Potassium	1.81	1.81	1.76	1.78
Sodium	0.33	0.34	0.33	0.34
Energy, Mcal/kg of DM				
NE_L_	1.70	1.70	1.70	1.72
NE_M_	1.90	1.90	1.90	1.92
ME	2.84	2.84	2.84	2.89

1 Unsupplemented treatment group (0 g of GML/d).

2 Low-dose treatment group (50 g of GML/d).

3 High-dose treatment group (150 g of GML/d).

4 Concentrate mix A contained 34.2% soybean meal, 26.3% fine-ground corn grain, 25.4% ground soybean hulls, 3.85% dried blood meal, 2.52% rumen-protected fat (Energy Booster 100; Milk Specialties Global), 1.94% limestone, 1.55% potassium bicarbonate, 1.14% sodium bicarbonate, 0.81% salt, 0.58% dicalcium phosphate, 0.51% lysine (Smartamine ML; Adisseo USA Inc.), 0.51% vitamin mix (Purina Animal Nutrition), 0.30% magnesium oxide, 0.27% methionine (Smartamine M; Adisseo USA Inc.), and 0.12% selenium.

5 Concentrate mix B contained 50.0% ground corn and 50.0% soybean meal.

6 Concentrate mix C contained 39.0% ground corn, 39.0% soybean meal, and 22.0% GML.

7 Concentrate mix D contained 17.0% ground corn, 17.0% soybean meal, and 66.0% GML.

Feed samples were analyzed for DM, CP, soluble protein, ash-free neutral detergent fiber (**aNDFom**), ADF, total digestible nutrients, ash, ether extract (**EE**), and lignin (Cumberland Valley Analytical Services, Waynesboro, PA). During wk −1 and 3, milk was sampled 3 times per day for 3 d. Milk samples were analyzed for fat, true protein, lactose, MUN, and TS concentrations using Fourier transform infrared spectroscopy and SCC by flow cytometry (Dairy One, Ithaca, NY). Milk samples for analysis of FA composition were composited based on milk fat yield to represent wk −1 and 3, relative to start of treatment. Samples were centrifuged at 17,800 × *g* for 30 min at 4°C and fat cakes were collected and stored at −80°C until the lipids from the fat cakes were extracted. The total lipid of the fat cake (320 ± 10 mg) was extracted using an n-hexane/isopropanol solution (3:2, vol/vol) (Lock et al., 2013). The FA composition was determined by gas-liquid chromatography, performed on a GC system-8890 (Agilent Technologies, Palo Alto, CA). Further details on the GC analysis methods are described in Gutierrez-Oviedo et al. (2024).

Enteric CH_4_, CO_2_, and H_2_ emissions were estimated during 4 d of wk −1 and 3 using the GreenFeed system (C-Lock Inc., Rapid City, SD). A randomized subset of 36 cows (12 cows/treatment) were used for gas measurements. Before the start of the experiment, cows were trained so they would become acclimated to the GreenFeed units. Spot gas sampling started at 0000, 0800, and 1600 h (d 1); 0030, 0830, and 1630 h (d 2); 0130, 0930, and 1730 h (d 3); and 0200, 1000, and 1800 (d 4) during wk −1. Spot gas sampling started at 0000, 1000, and 1600 h (d 1); 0030, 1000, and 1630 h (d 2); 0130, 1000, and 1730 h (d 3); and 0200, 1000, and 1800 (d 4) during wk 3. Cows were sampled in the same order. A pellet cattle feed (4-Square Stocker Grower Textured Beef Cattle Supplement; Purina Animal Nutrition LLC, Shoreview, MN) was used as a bait feed. Each sample collection took 5 to 7 min with an additional 2 min for background measurements.

Blood for plasma collection was collected from all cows once via caudal vein venipuncture during wk −1 and 3 at ∼0500 h. Upon collection, blood samples were immediately placed on ice for ∼30 min, followed by centrifugation at 2,171 × *g* for 20 min at 4°C. The plasma samples were stored at −80°C until analysis. Plasma total FA (HR series NEFA-HR no. 999–34691, 995–34791, 991–34891, 993–35191, Wako Chemicals USA Inc., Richmond, VA) and glucose (Autokit Glucose no. 997–03001, FUJIFILM Medical Systems USA) were quantified in duplicate according to the manufacturer's instructions.

The yields of 3.5% FCM, ECM, and milk components were calculated using milk yields and component concentrations from each milking, summed for a daily total, and averaged for each collection period, as follows: 3.5% FCM = [(0.4324 × kg of milk) + (16.216 × kg of milk fat)]; and ECM = [(0.327 × kg of milk) + (12.95 × kg of milk fat) + (7.65 × kg of milk protein)] (Martin et al., 2021). Somatic cell score was calculated from SCC for statistical analysis using a logarithmic transformation, where log_2_ (SCC/100,000) + 3 (Ali and Shook, 1980). The feed efficiencies for milk yield, 3.5% FCM, and ECM production were calculated as the ratio of milk yield, FCM, or ECM in relation to DMI. Each week's gas emission samples were summed to determine total gas production estimates for the sampling period. Gas emissions were analyzed for intensity by calculating gas production per kilogram of milk yield as well as per kilogram of ECM and 3.5% FCM. Gas yield was defined as the total production of the gas per unit of DMI (g gas production/kg DMI).

Statistical analyses were carried out using the mixed model procedure of SAS (v9.4, SAS Institute Inc., Cary, NC) according to the following model:

*Y_ijk_* = *μ* + *C_i_* + *T_j_* + *D_k_* + *T_j_* × *D_k_* + *PAR* + *pVar_i_* + *e_ijk_*,

where *Y_ijk_* = dependent variable; *μ* = overall mean effect for the measure; *C_i_* = random effect of cow (*i* = 1 to 42); *T_j_* = fixed effect of treatment (*j* = 1 to 3); *D_k_* = fixed effect of day (*k* = 1 to 21); *T_j_* × *D_k_* = fixed effect of the interaction between treatment and day; *PAR* = parity used as a covariate; *pVar_i_* = baseline measurement for each response variable used as a covariate; *e_ijk_* = the residual error. After assessing 5 distinct covariance structures (variance components, first-order autoregressive, unstructured, compound symmetry, and first-order ante dependence), the most appropriate covariance structure for each variable in the repeated measures analysis was chosen. The selection process involved identifying the structure with the lowest Akaike's information criterion coefficient for subsequent analysis. A post hoc Tukey test was employed for multiple comparisons to compare differences within each time point. The model was used to evaluate production responses, gas measurements, and plasma metabolites. Observations were deemed as outliers if studentized residuals >3.0 or <−3.0. Normality of the residuals was checked with normal probability and box plots and homogeneity of variances with plots of residuals versus predicted values to ensure no violation of model assumptions. The LSM comparisons were performed using preplanned contrasts of interest (LD vs. CON and HD vs. CON) using adjusted *P*-values. Main effects were declared significant at *P* ≤ 0.05 and trending toward significance at 0.05 < *P* ≤ 0.15. Results are expressed as LSM, unless otherwise noted.

Treatments had no effect on milk yield or DMI (Table 2). Additionally, ECM yields, milk lactose contents and yields, milk solids contents and yields, and milk MUN were not modified by treatment (Table 2). The HD cows had greater milk fat contents (4.22 vs. 4.01%) and yields (1.76 vs. 1.62 kg/d) compared with CON cows (*P* = 0.02 and *P* = 0.05, respectively; Table 2). The LD and HD cows had higher concentrations of de novo FA and lauric acid as well as greater yields of de novo and mixed FA and lauric, myristic, palmitic, and stearic acid, relative to CON (*P* ≤ 0.01; Table 3). Short- and medium-chain FA make up around 40% of the FA in milk (Chilliard et al., 2000). These FA originate from de novo FA synthesis in the mammary gland and are needed to maintain milk fat fluidity and form milk triacylglycerols (Palmquist and Jenkins, 1980). Of the short-chain and medium-chain FA present in milk, lauric and myristic FA have the highest transfer efficiency rates at 67 and 83%, respectively (Kadegowda et al., 2008). Their results align with our findings, as we observed an increase in lauric acid contents and yields with dietary GML supplementation in both the LD and HD treatment groups. As previously discussed, lauric acid has exhibited various anti-inflammatory, anti-viral, and anti-bacterial properties. Enhancing the contents of lauric acid in dairy products is one potential strategy to boost lauric acid consumption in human populations, potentially leading to long-term improvements in human health.

**Table 2:** Effects of dietary supplementation with glycerol monolaurate on productive performance, milk composition, and feed efficiency

Variable	Treatment			SEM^4^	*P*-value		
	CON^1^	LD^2^	HD^3^		Treatment	CON vs. LD	CON vs. HD
Productive performance							
Milk yield, kg/d	41.9	42.2	42.9	0.56	0.41	0.76	0.22
3.5% FCM,^5^ kg/d	44.8	45.7	46.6	0.76	0.30	0.43	0.13
ECM,^6^ kg/d	45.3	45.8	46.5	0.71	0.51	0.62	0.26
DMI, kg/d	30.4	30.6	29.8	0.37	0.25	0.69	0.26
BW, kg	751	757	748	5.16	0.43	0.44	0.67
BCS	3.10	3.05	3.12	0.04	0.47	0.43	0.72
Rumination, min/d	530	532	545	7.96	0.37	0.87	0.20
Plasma glucose, mg/dL	74.4	70.3	73.5	2.23	0.37	0.19	0.77
Plasma nonesterified FA, μmol/L	91.9	91.8	102.9	10.4	0.68	0.99	0.47
Milk composition, %							
Fat	4.01	4.15	4.22	0.07	0.16	0.20	0.05
Protein	3.49	3.48	3.42	0.02	0.02	0.71	0.01
Lactose	4.83	4.82	4.82	0.02	0.99	0.88	0.91
Solids	13.3	13.4	13.4	0.08	0.39	0.20	0.26
SCS^7^	1.95	1.78	2.06	0.26	0.75	0.66	0.79
MUN, mg/dL	10.5	11.0	10.9	0.29	0.52	0.27	0.41
Milk solids, kg/d							
Fat	1.62	1.71	1.76	0.03	0.01	0.21	0.02
Protein	1.52	1.46	1.46	0.02	0.07	0.04	0.05
Lactose	2.05	1.99	2.01	0.04	0.48	0.24	0.41
Feed efficiency							
Milk yield/DMI	1.38	1.39	1.42	0.02	0.25	0.75	0.13
ECM/DMI	1.50	1.51	1.57	0.03	0.21	0.84	0.12
FCM/DMI	1.48	1.51	1.57	0.03	0.13	0.64	0.06

1 Unsupplemented treatment group (0 g of GML/d).

2 Low-dose treatment group (50 g of GML/d).

3 High-dose treatment group (150 g of GML/d).

4 Pooled SEM.

5 3.5% FCM = (0.4324 × kg of milk yield) + (16.216 × kg of milk fat yield).

6 ECM = (0.327 × kg of milk yield) + (12.95 × kg of milk fat yield) + (7.65 × kg of milk protein yield).

7 SCS = log_2_ (SCC/100,000) + 3.

**Table 3:** Effects of dietary supplementation with glycerol monolaurate on milk fatty acid (FA) concentrations (g/100 g of milk fat) and yields (g/d)

Variable	Treatment			SEM^4^	*P*-value		
	CON^1^	LD^2^	HD^3^		Treatment	CON vs. LD	CON vs. HD
Fatty acids, %							
De novo^5^	23.0	23.7	23.7	0.28	0.01	0.01	0.01
Mixed^6^	32.6	32.9	33.1	0.48	0.64	0.59	0.35
Preformed^7^	30.2	29.1	28.9	0.51	0.03	0.03	0.01
C12:0	3.98	4.32	4.78	0.10	<0.01	<0.01	<0.01
C14:0	11.5	11.6	11.4	0.12	0.25	0.40	0.45
C16:0	31.0	31.3	31.4	0.45	0.65	0.46	0.39
C18:0	6.46	6.60	6.59	0.21	0.78	0.52	0.54
C18:1 *cis*-9	13.5	12.6	12.7	0.21	0.01	0.00	0.01
C18:2 *cis*-9,*cis*-12	2.00	1.94	1.82	0.03	0.00	0.12	<0.01
C18:2 *cis*-9,*trans*-11	0.31	0.30	0.28	0.01	0.07	0.36	0.03
C18:3 *cis*-9,*cis*-12,*cis*-15	0.29	0.28	0.26	0.00	0.01	0.49	0.00
Fatty acids, g/d							
De novo^5^	367	409	419	10.9	<0.01	<0.01	<0.01
Mixed^6^	529	564	585	18.4	0.02	0.05	<0.01
Preformed^7^	487	501	504	9.40	0.40	0.27	0.21
C12:0	63.9	74.3	84.4	2.60	<0.01	<0.01	<0.01
C14:0	185	200	202	5.37	0.01	0.01	<0.01
C16:0	502	538	557	17.3	0.01	0.04	<0.01
C18:0	102	114	117	4.30	<0.01	0.01	<0.01
C18:1 *cis*-9	218	216	218	4.63	0.95	0.77	0.95
C18:2 *cis*-9,*cis*-12	32.5	33.2	31.7	0.66	0.28	0.46	0.42
C18:2 *cis*-9,*trans*-11	5.01	5.03	4.85	0.17	0.71	0.91	0.53
C18:3 *cis*-9,*cis*-12,*cis*-15	4.68	4.88	4.57	0.14	0.26	0.32	0.58

1 Unsupplemented treatment group (0 g of GML/d).

2 Low-dose treatment group (50 g of GML/d).

3 High-dose treatment group (150 g of GML/d).

4 Pooled SEM.

5 De novo FA originated from mammary de novo synthesis (<16C).

6 Preformed FA originated from extraction from plasma (>16C).

7 Mixed FA originated from both sources (C16:0 plus C16:1 *cis*-9).

Both lauric and palmitic acids stimulate mammary gland cells to secrete FA, which are then incorporated into milk as triacylglycerol (Hansen and Knudsen, 1987). As such, researchers have predicted the dietary supplementation of short-chain and medium-chain FA may increase milk fat content and yields (Vyas et al., 2012). This explains the increases in milk fat contents and yields we observed with the HD treatment group. The HD cows tended to produce more 3.5% FCM, relative to CON (46.6 vs. 44.8 kg/d; *P* = 0.13; Table 2). The increases in milk fat contents and yields in the HD cows were accompanied accordingly by tendencies for improved feed efficiency (i.e., kg of milk, 3.5% FCM and ECM per unit of DMI), relative to CON (*P* = 0.13, 0.06, and 0.12, respectively; Table 2).

Milk protein contents were lower for HD cows (3.42 vs. 3.49%, *P* = 0.01; Table 2). Milk protein yields were also lower in LD and HD, relative to CON (1.46, 1.46, and 1.52 kg/d, respectively; *P* = 0.04 and 0.05; Table 2). Milk protein yields were likely lower in the HD group due to the lower CP content in the diets, according to the analysis (15.4% vs. 16.2% CP; Table 1). Additionally, short-chain, medium-chain, and unsaturated FA have been known to be more toxic to microbes in the rumen than long-chain, saturated, or esterified FA (Henderson, 1973). Milk protein contents and yields in cows fed LD or HD may have been lower due to the antimicrobial properties that GML possesses, suppressing the growth of a wide range of gram-positive and gram-negative bacteria (Schlievert and Peterson, 2012). As such, the monoester may have reduced microbial protein synthesis and flow to the small intestine. Moreover, researchers have attributed the reduced milk protein contents that result from dietary fat supplementation to the fat affecting the digestion and absorption of protein, resulting in a reduced AA supply to the mammary gland and a reduction in milk protein synthesis (Wu and Huber, 1994).

No modifications were observed in CH_4_ production, CH_4_ intensity, or CH_4_ yield. Similarly, no modifications were observed in CO_2_ production, CO_2_ intensity, or CO_2_ yield. Moreover, no changes in H_2_ production, intensity, or yield were observed. It is possible there was no observed impact on gas production, yield, or intensity due to the 3-wk duration of the treatment period. This is a short feeding period and a lower dose when compared with a 10-wk study supplementing cows with an alternating treatment of Agolin Ruminant at 0.17 g/kg DM and lauric acid at 65 g/kg of DM, where they observed a 20% decrease in CH_4_ production (Klop et al., 2017). It has been suggested the adaptation period of the rumen microbiome can last several days to weeks (Schären et al., 2017). It is possible that 3 wk was an insufficient amount of time or the dosage of GML was too low to alter the rumen microbiome. Protozoa are significant contributors to methanogenesis in the rumen; they produce ample H_2_, most of which is later converted to CH_4_ by methanogens hosted by the protozoa (Morgavi et al., 2010). Ciliate protozoa contribute to a disproportionate amount of the methanogenesis happening in the rumen. It is estimated their activity can account for up to 37% of total CH_4_ production (Finlay et al., 1994). If there was insufficient time or quantity of GML supplemented in the diet for the ruminal protozoal populations to shift, it is unlikely a reduction in CH_4_ production is observed.

In acknowledgment of the design limitations of our experiment, it is important to note that we used doses of GML lower than what has been reported by Klop et al. (2017) when evaluated on the basis of lauric acid alone and with the intent to not compromise DMI. Indeed, Klevenhusen et al. (2009) also evaluated high GML levels (50 g/kg of DM); however, we must realize that supplemental commercial fats in the diet typically range between 0.5 and 1.5% of ration DM (5 to 15 g/kg of DM). Moreover, these fats are frequently palmitic, stearic, and oleic acids. Although feeding GML at higher feeding levels may have reduced methane, such findings would fall short of standard feeding practices for lipids. We also recognize the limitation of using a limited number of animals with a short treatment duration, which may have resulted in less reliable daily gas emission estimates, as there are fewer total spot gas measurements. This said, we conclude that under the present conditions of this study, dietary supplementation of GML can be used to modify milk production, demonstrating increases in milk fat contents, milk fat yields, de novo and mixed FA contents, and FA yields, but decreases in protein contents and yields at a feeding level of 150 g/d. Supplementation at a more conservative dose of 50 g/d can be used to increase de novo FA contents and yields as well. However, GML feeding within our defined experimental conditions did not modify enteric CH_4_ production in dairy cattle. Future work should investigate the effects of dietary GML supplementation using various treatment durations, how GML modifies the rumen microbiome, and different dosage effects of GML. This information would help to optimize the usage of GML in commercial situations.
